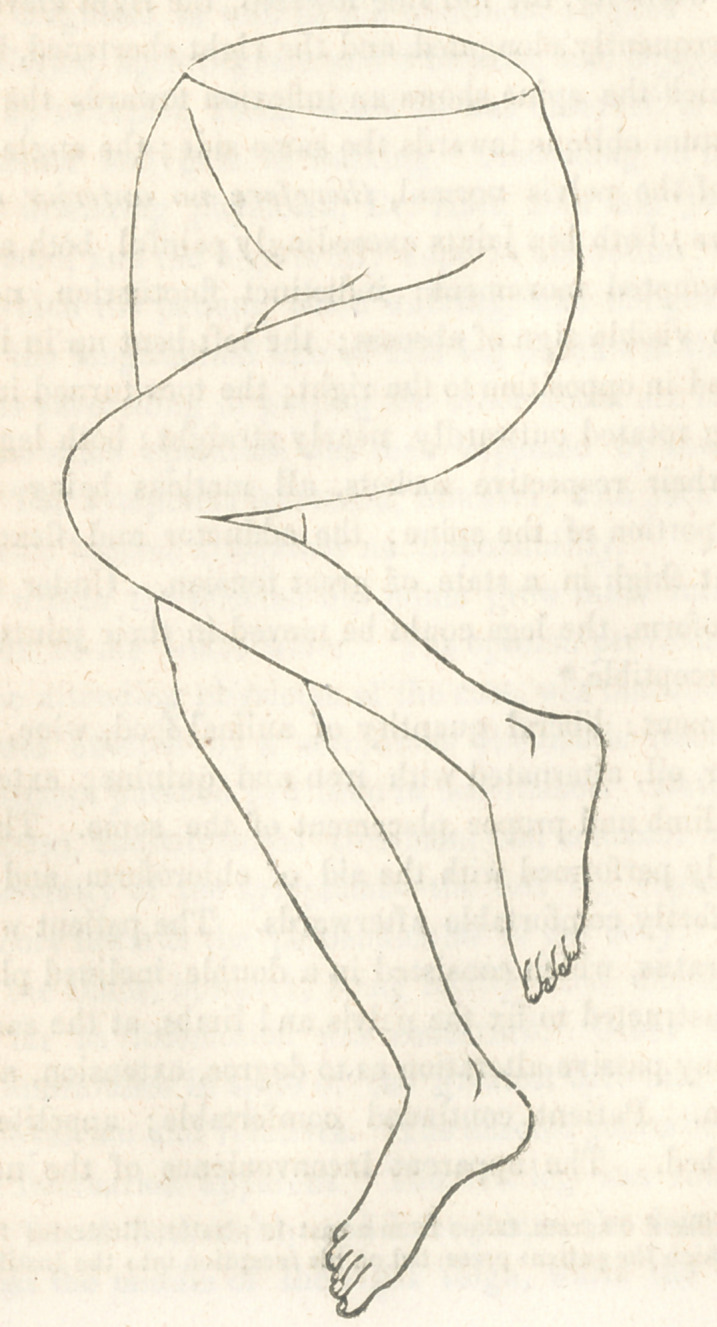# A Case of Caries of Both Hip Joints, Etc.

**Published:** 1854-04

**Authors:** A. Stone

**Affiliations:** Brooklyn, N. Y.


ARTICLE II.—*A Case of Caries of both Hip Joints—Uncertainties of Diagnosis—Memoranda of Treatment.* By A. STONE, M D.

BUT few men are endowed with that genius and that penetratin power of observation and combination, indispensably requisite for a revolutionary movement in science, and but few have inherited the lot of being placed in such an advantageous position of developing and applying those gifts of benevolent nature. At any rate, the writer hopes not to range under either category; he may, however, become the instrumentality for the advancement of science, by serving as an exponent of the scientific inquiries, experiments and results, made and attained by others.

The polite invitation extended to me by Drs. Bauer and Barthelmess, physicians to the Orthopedic Institution of this city, has afforded the writer the rare opportunity of daily witnessing, for several months past, cases under treatment, developing some of the most instructive principles of pathology and cure pertaining to deformities and joint affections. From among the number of highly interesting cases, the communication of the following, it is thought, may be very acceptable to the press on account of its instructive character in diagnosis, as well as the treatment adopted:

William Carter, aged 5 years and 8 months, had enjoyed excellent health up to April, 1852, when he was observed to assume a peculiar manner and gait in walking. According to the statement of his attending physician, Dr. Kalt, both hip joints were immovable, sore, and the legs firmly kept in abduction; in consequence of which the patient, when walking, was obliged to turn his body on the longitudinal axis of that leg which was then on the ground, thus succeeding in putting the other about six inches forward, and the same operation was then repeated by the first leg and so on. The symptoms increased, however, with such rapidity, that locomotion became altogether an impossibility.

The pain within the affected hip joints grew more intense, and the separation of the limbs wider. The opinion previously entertained by the attending physician of the case, was that the synovial membrane was affected by a scrofulous rheumatic inflammation, and the treatment pursued had been in accordance. Baths, superficial derivation, constitutional treatment and constant rest, alleviated the severity of the symptoms—the left leg, especially, approaching more towards the perpendicular of the body. The new position of the limbs, however, being totally fixed, afforded no advantage as far as locomotion was concerned. Other symptoms made their appearance in spite of the greatest care and attention from both physician and relatives. The affected joints became distended, and fluctuation apparent. The left leg was rotated outwardly and flexed both in hip and knee joints, the latter resting on and across the middle of the right thigh, while the right leg remained almost straight, but at the same time being rotated outwardly. In this state the patient was received into the Orthopedic Institution.

The case-book of this establishment contains the following notes on the case: Great pallor and emaciation; hectic fever, appetite good, digestion regular; dermoid surface dry and sluggish; sleep undisturbed; pain in the affected joints when moved, but not otherwise; general strumous appearance; induration and enlargement of lymphatic glands; discharge from one ear; thorax rather flat; auscultation and percussion discovered no abnormal sound; pelvis in a state of declivity, the left side lowered, the right elevated; the left leg consequently elongated, and the right shortened, in conformity with which the spine shows an inflexion towards the left, and the rima natum oblique towards the same side; the angle of the inclination of the pelvis normal, *therefore no anterior curvature of the spine;* both hip joints exceedingly painful, both as to pressure and attempted movement; indistinct fluctuation, no redness or heat, no visible sign of abscess; the left bent up in hip and knee joint, and in opposition to the right; the toes turned inwardly; the right leg rotated outwardly, nearly straight; both legs immovable within their respective sockets, all motions being made in the lumber portion of the spine; the adductor and flexor muscles of the right thigh in a state of great tension. Under the influence of chloroform, the legs could be moved in their joints, but no crepitus perceptible.*

Treatment: liberal quantity of animal food, wine, ale, brandy, cod-liver oil, alternated with iron and quinine; extension of the affected limb and proper placement of the same. This operation was easily performed with the aid of chloroform, and the patient felt perfectly comfortable afterwards. The patient was placed in his apparatus, which consisted in a double inclined plane, ingeniously constructed to fix the pelvis and limbs, at the same time allowing any passive alteration as to degree, extension, abduction, or adduction. Patient continued comfortable; appetite and sleep undisturbed. The apparent inconvenience of the new position,

* The affixed diagram, taken from a cast in plaster, illustrates the peculiar deformity which the patient presented on his reception into the Institution.

did not have the least unfavorable effect upon his general health, on the contrary he continued to improve rapidly considering his past enfeebled state of body.

The opening of the joints for the purpose of assisting nature more speedly to throw off the morbid matter and thereby reestablish a recuperative action was at this time, (the time of stretching the limb) a mooted question by the able surgeons of the institution, but postponed on account of the probability of the purulent matter becoming absorbed, after the parent cells having bursted and re-arranged to other organisation capable of absorption, — an opinion sustained by able pathologists — Professors Alonzo Clark, Paget, Miller and others — and indeed this hope, in this case at least, seemed to become realized, for the fluctuation of the joints became much less perceptible during the subsequent weeks of treatment; but then it increased again and so rapidly in the left hip joint that the matter could be felt quite distinctly. At this period of the case, an opinion was expressed by the surgeons of the institution, that a rupture of the synovial membrane had taken place, and a free opening of the abscess, and an enlargement of the spontaneous opening of the capsular ligament was rationally indicated. Consequently in presence of Drs. Sayre, Surgeon of Bellvue Hospital, N. Y., Kolt, Stoltz, myself and others, a large incision was made, the patient being under the influence of chloroform.

A large quantity of pus escaped. By digital examination, the great extent of the ulceration as well as the perforation of the joint was ascertained, being the posterior wall of the capsular ligament. A minute examination discovered the joint to be in the acetabulum, although the head of the joint was denuded and the connecting ligament destroyed, the acetabulum not being increased in size.

Injections of warm water was freely made, washing out both the cavity of the abscess and the joint, and finally filled with lint; the opening in the capsular ligament being large enough to allow the free discharge of the morbid contents of the joint. The patient having lost but a few drops of blood rallied very soon and expressed himself as being greatly relieved and comfortable. But a slight operation followed that operation both locally and constitutionally, healthy granulations and pus made their appearence and in six weeks the whole cavity was filled and consolidating, the patient at the same time improving generally.

Six weeks after the operation of opening the first or left hip joint, soreness and apparent inflammation was manifested in the right joint also: the patient was troubled with chills and occasional exacerbations of fever and dryness of skin and other constitutional symptoms, which together with the fixed condition of the joint warranted the unanimous conclusion in full consultation with the surgeons, called in for that purpose, that the cavity of the joint was filled with purulent matter and the whole integrity of the joint seriously implicated. Although fluctuation could not be distinctly made out, the various considerations induced to a certain diagnosis by first exploring with a trocar. This was introduced superiorly and posteriorly of the great trochauter and under the joint at the posterior circumference, between the cotyloid cartilage and caput femoris. A column of thick pus immediately ascended the canular, which was now removed and an incision made at the same place about 6½ inches long, and the articular sack opened along the posterior hemisphere. About 6 to 9 ounces of consistent purulent matter escaped. The articular cavity, in as far as it could be ascertained, felt rough, both the greater part of the acetabulum and the posterior part of the femoral head being denuded; the ligamentum teres however being not destroyed. Caput femoris was found to be in its normal place, and the leg could not be rotated inwardly. I need hardly state, that crepitus was subsequently heard and felt. After the patient had been properly dressed and fixed in a leather splint, he revived and felt comfortable and relieved. The third day after the operation, the patient was doing well, cheerful, and to all appearance sustained the effects of the operation exceedingly well.

This is a history of a case, and a treatment no doubt involving questions of the greatest interest for the profession.

My private practice has not yet afforded me an opportunity of testing the new treatment, but the cases I have observed in the *Orthopedic Institution* in this city, tend to prove its soundness and efficacy. There are many surgeons who no doubt forcibly condemn the practice adopted by the surgeons of this institution, but they will fail of overruling the arguments and results in favor of freely opening ulcerated joints, while on the other hand, arguments principally consisting in the much dreaded entrance of air into the articular cavity is anything but conclusive.

There are certainly a good number of cases on record,—lately increased by 3 others observed by Dr. Judkins, (Ohio Medical and Surgical Journal, No. 3, vol. VI.) which terminated fatally after the free opening had been made, but it remains yet to be decided whether the operation or bad food, air, want of attention and other necessaries, or the long postponement, are not chargeable with the health of the patient. Dr. Judkins himself refers to the preponderance of errors peculiar in the hospitals of Paris, frustrating but too often the results of many skilfully performed operations, and by the greatest masters of surgical art, and exactly the same we see in Bellvue Hospital. On the other hand, we hear of successful opening of the joints both from British and German Hospitals, especially of John Gay, Esq. Surgeon of the Royal Free Hospital, London. At all events the results of the old school in reference to the treatment of ulcerative diseases of joints, are of so inferior an order that the new mode has at least a right to be fairly tested. Competent men, unprejudiced by any established opinion, may then decide whether the treatment advocated and practiced by Dr. Bauer at the *Orthopedic Institution* in ulcerations of joints in general, and especially of the hip joint, is correct and reliable or not.

Brooklyn, N. Y. March 4, 1854.

## Figures and Tables

**Figure f1:**